# How Health Behaviors Relate to Academic Performance via Affect: An Intensive Longitudinal Study

**DOI:** 10.1371/journal.pone.0111080

**Published:** 2014-10-29

**Authors:** Lavinia Flueckiger, Roselind Lieb, Andrea H. Meyer, Jutta Mata

**Affiliations:** 1 University of Basel, Department of Psychology, Division of Clinical Psychology and Epidemiology, Basel, Switzerland; 2 Center for Adaptive Rationality (ARC), Max Planck Institute for Human Development, Berlin, Germany; Tokyo Institute of Technology, Japan

## Abstract

**Objective:**

This intensive longitudinal study examined how sleep and physical activity relate to university students’ affect and academic performance during a stressful examination period.

**Methods:**

On 32 consecutive days, 72 first-year students answered online questionnaires on their sleep quality, physical activity, positive and negative affect, learning goal achievement, and examination grades. First-year university students are particularly well-suited to test our hypotheses: They represent a relatively homogeneous population in a natural, but controlled setting, and simultaneously deal with similar stressors, such as examinations. Data were analyzed using multilevel structural equation models.

**Results:**

Over the examination period, better average sleep quality but not physical activity predicted better learning goal achievement. Better learning goal achievement was associated with increased probability of passing all examinations. Relations of average sleep quality and average physical activity with learning goal achievement were mediated by experienced positive affect. In terms of day-to-day dynamics, on days with better sleep quality, participants reported better learning goal achievement. Day-to-day physical activity was not related to daily learning goal achievement. Daily positive and negative affect both mediated the effect of day-to-day sleep quality and physical activity on daily learning goal achievement.

**Conclusion:**

Health behaviors such as sleep quality and physical activity seem important for both academic performance and affect experience, an indicator of mental health, during a stressful examination period. These results are a first step toward a better understanding of between- and within-person variations in health behaviors, affect, and academic performance, and could inform prevention and intervention programs for university students.

## Introduction

Starting university is an academically and socially challenging transition; the university years are often considered the most demanding learning period in many people's lives. About a third of students entering university-level education leave without a degree [1]. Concerning study duration, on average students take a good semester longer to graduate than recommended [2]. The consequences of university dropout or prolonged study duration include increased education costs [3] and unfulfilled dreams—for example, not having the qualifications to work in one's chosen profession or earning a lower salary [1].

Therefore, identifying behaviors that can help young adults to achieve their academic goals is of considerable importance. This prospective longitudinal study investigated whether health behaviors, such as sleep and physical activity, are related to academic performance in young adults. Specifically, we examined both, average values across the entire study period as well as day-to-day dynamic relations. We tested how sleep quality and physical activity were related to learning goal achievement and, in turn, whether average learning goal achievement predicted success in year-end examinations. Furthermore, we tested whether positive and negative affect mediated the relations of sleep quality and physical activity with learning goal achievement.

### Health behaviors and academic performance

Health behaviors such as sleep and physical activity have been associated with increased cognitive performance and better grades [4], [5]. A meta-analytic review has demonstrated that better overall sleep quality and longer sleep duration in children and adolescents are related to better grades [4]. Additionally, a review by Curcio, Ferrara, and De Gennaro [6] has shown that poor overall sleep quality in students from school to university is associated with impairment of cognitive performance, reduced learning behavior, and weaker academic performance. Likewise, Ahrberg, Dresler, Niedermaier, Steiger, and Genzel [7] found that higher sleep quality prior to examinations (but not during the semester or after the examination period) was linked to better academic performance in a sample of university students.

Concerning the role of physical activity, it has consistently been shown that acute aerobic activity is related to improvements in cognitive performance [5]. Importantly, high levels of regular physical activity have been associated with better grades [8]–[10] and higher self-perceived overall academic performance in children and adolescents [11], [12]. Importantly, the existing literature has focused on the association of sleep and physical activity with academic performance on the between-person level.

### Affect as a potential mechanism underlying the association between health behaviors and academic performance

Several physiological mechanisms have been suggested as underlying the relation between health behaviors such as physical activity and cognitive performance, including improvement in oxygen consumption [13], increased task-related brain activity [14], [15], and increased brain volume in the prefrontal and temporal cortices in older adults [16]. Patterns of activation in prefrontal cortex have also been suggested as an underlying mechanism for the relation between sleep and cognitive performance [17]. Potential psychological mechanisms have received less attention.

One plausible psychological mechanism is affect: Positive affect is associated with successful outcomes in various life domains, including health and academic performance [18], [19]. Specifically, affect has been demonstrated to influence different aspects of learning behavior, motivation [20] and achievement [21]. In recent years, researchers have started to test the link between affect and learning behavior at the within-person level. When immediate feedback is absent, as is the case when students study for a major exam over several weeks, current affect may be used as input for setting learning goals [22] as well as on goal achievement and progress judgment [23]. This idea is in line with the mood-as-information model by Schwarz and Clore [24], which claims that current affect serves as a source of information for judgments. Richard and Diefendorff [22] observed that higher positive affect in university students predicted higher learning goals the next day, whereas higher negative affect predicted lower learning goals the next day. Likewise, in a sample of employees, Miner and Glomb [25] showed that periods of positive affect were associated with periods of improved task performance.

The empirical evidence also indicates that sleep and physical activity influence affect on the between- and within-person level. A meta-analysis and reviews have shown that sleep deprivation adversely impacts affect [26]–[28]. As a potential underlying mechanism for the relation between sleep and affect, it has been suggested that sleep deprivation leads the prefrontal cortex region to inhibit the amygdala, a brain structure that is crucial for the generation and recognition of affect [29]. However, few studies have demonstrated that increased sleep quality predicts increased positive affect on a between-person [30] or a within-person level [31], [32]. Other studies have shown that being physically active predicts higher positive and lower negative affect [33], [34], and meta-analyses have suggested that acute as well as regular aerobic exercise produces increases in positive affect [35], [36]. For these beneficial effects of physical activity on affect, several potential psychological and physiological mechanisms have been discussed, including the distraction hypothesis, self-efficacy theory, as well as the endorphin and monoamin hypothesis [37].

Importantly, young adulthood is not only a time of academic challenges, but also a critical period for the onset of mental illnesses such as mood and anxiety disorders [38], [39]. Specifically, the first year of university is a critical time for the first occurrence of depressive symptoms [40]. Identifying behaviors that impact affect experience in this period is therefore particularly important. Given the previous findings on health behaviors influencing affect and on the involvement of affect in academic performance, it is plausible that health behaviors might influence academic performance through changes in affect.

### Academic performance

Academic performance in tertiary education is a construct that has been assessed on different dimensions, including intelligence, cognitive capacity, and examination success [41], [42]. However, examination success is the key requirement for continuation of studies and graduate employment [43] and therefore, examination success and grades are still the most widespread performance measures [44]. Research has shown that self-regulatory learning strategies [44], [45] as well as goal setting [46] predict better grades. Self-regulation may be particularly important for maintaining the motivation of learners pursuing long-term goals without immediate performance feedback - as is the case for students studying for major examinations [47]. Therefore, the achievement of students' self-set learning goals may predict examination success.

### Hypotheses

**Figure 1:**
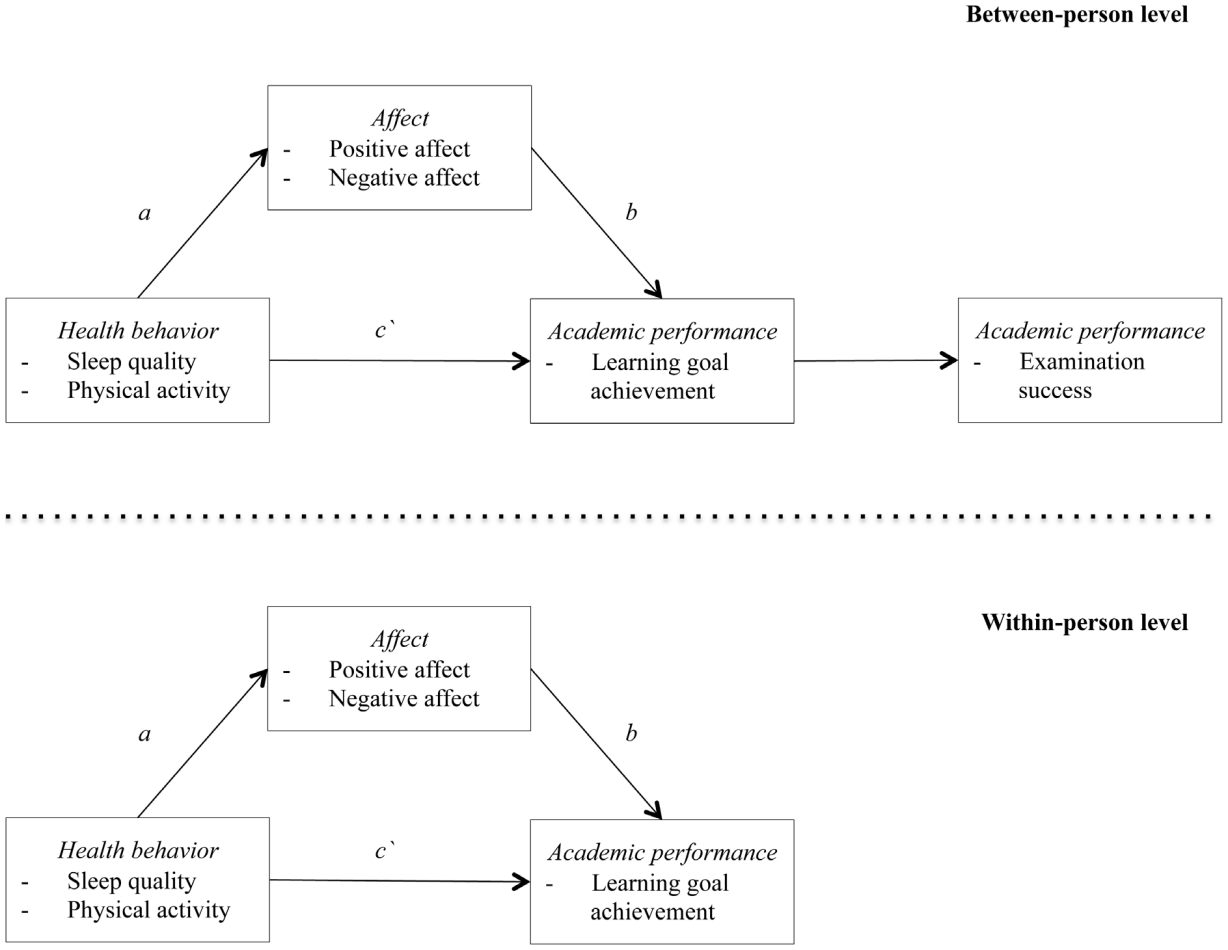
Mediation model of health behaviors on academic performance via affect on between- and within-person level.

To our knowledge, no previous study has combined health behaviors, affect, and academic performance within a single model and evaluated their relations on a between-person level as well as at the level of day-to-day dynamics. Based on the theoretical foundations, including direction of associations, reviewed above, we test the following hypotheses (see also Figure 1):

#### Between-person level

(1a) Better average sleep quality predicts better average learning goal achievement; higher average physical activity predicts better average learning goal achievement.

(1b) Average positive/negative affect mediates the relation between average sleep quality and average learning goal achievement (e.g., higher average sleep quality predicts higher positive affect which in turn predicts better average learning goal achievement). Likewise, average positive/negative affect mediates the relation between average physical activity and average learning goal achievement.

(1c) Students who on average report better learning goal achievement are more likely to pass their examinations.

#### Day-to-day level (within-person level)

(2a) On days on which students report higher sleep quality, they also report better learning goal achievement; on days on which students report higher physical activity, they also report better learning goal achievement.

(2b) Daily positive/negative affect mediates the relation between sleep quality and learning goal achievement as well as the relation between physical activity and learning goal achievement (e.g., on days with higher sleep quality, students experience higher positive affect which in turn predicts better learning goal achievement).

## Methods

### Design

Data were obtained from a prospective, intensive longitudinal survey. First-year psychology students from the University Basel, answered online questionnaires during 46 consecutive days over the year-end examination period from May to July 2011. The first 14 days involved examination preparation; the following 18 days were the examination period proper; and the last 14 days were the post-examination period. Because this article aims to understand behavior and experience during the preparation phase and to predict examination success, only data collected during the first 32 days were used in the present analyses. Participants sat six examinations that are obligatory for all first-year psychology students at the University of Basel. Importantly, students failing at least one of these examinations more than once are excluded from studying psychology at any Swiss university.

### Participants

First-year university students were chosen as participants for this study because for several reasons they are a particularly well-suited population to test our hypotheses: For one, they simultaneously have to deal with the same stressors, such as final examinations, thus they are in a natural, but relatively controlled setting. Second, most students are of similar age and education background, thus, they represent a comparably homogeneous population. Third, all university students receive Internet access through the university, thus conducting an online study does not lead to selection biases. The original sample consisted of 82 first-year psychology students from the University of Basel, who volunteered to participate in the study (142 students were enrolled as first year psychology students at the University of Basel at the time). Five participants did not take the final examinations and an additional five participants did not state their examination grades. These ten participants were excluded from the analyses, leaving a final sample of *N* = 72 (see Table 1 for sample characteristics). The ten excluded participants did not significantly differ from the 72 remaining participants in any of the variables of interest. There were no significant differences between men and women in any of the sample characteristics reported in Table 1 except for negative affect. Women reported higher negative affect compared to men *M* = 2.74, *SD* = 0.95 versus *M*  = 1.94, *SD*  = 0.70, *t*(11.23)  = 2.85, *p* = .016. The 72 participants completed daily questionnaires and delivered a total of 2111 responses over the 32 days, giving *M* = 29.3 completed questionnaires per person (*SD*  = 5.04; range: 8–32 questionnaires per person) which represents a compliance rate of 92%.

**Table 1:** Sample characteristics.

Characteristic	Total sample (*N* = 72)
Gender (women %)	70.1%
Age (median in years)	21.0
Number of semesters studied (median)	2.0
Learning goal achievement (*M, SD*)	2.2 (1.1)
Sleep quality (*M, SD*)	3.0 (0.8)
Physical activity (median)	180
Positive affect (*M, SD*)	4.2 (1.6)
Negative affect (median)	2.3

*Note*. *Learning goal achievement* was rated on a 5-point Likert scale from 0 (not at all) to 4 (completely); *sleep quality* was rated on a 4-point Likert scale from 1 (very bad) to 4 (very good); minutes of mild, moderate and strenuous *physical activity* were converted into metabolic equivalents; experiencing *positive and negative affect* was rated on a 7-point Likert scale from 1 (not at all) to 7 (extremely).

### Procedure

The ethics committee “Ethikkommission Nordwest- und Zentralschweiz (EKNZ)” approved the current study (Ref.Nr. EK: 48/11). Participants were recruited through announcements during first-year psychology lectures at the University of Basel. Interested students received an email with a link to the website of the online entry questionnaire, where they were given detailed information about the study. After giving written consent, participants received an email every day at 5 pm for 32 consecutive days. The email contained a link to that day's online questionnaire. At the end of the examination period, they completed an online exit questionnaire.

In each day's questionnaire, participants were asked to enter their personal code (made up of letters from their mother's first name, maiden name, and birthday). This code was used to link each participant's questionnaires. Participants were never asked to enter any information that would make it possible to identify them; responses to the questionnaires and email addresses could not be linked. On average, answering one daily questionnaire took 4.1 (*SD*  = 4.3) minutes. Students could choose between receiving financial compensation of up to 50 Swiss Francs, corresponding to approximately 55 US-Dollars, for participation or equivalent course credits in psychology; the exact level of compensation was dependent on the number of questionnaires completed.

### Measures

#### Entry and Exit Questionnaire

*Sociodemographic variables* were assessed in the entry questionnaire (see Table 1). The measure of *academic performance* on the between-person level, examination success, was calculated from the grades of the six final examinations that participants took. Participants reported the grades of these independent examinations in the exit-questionnaire. Internal consistency reliability for the grades of these six examinations was Cronbach's α = .89. It is important to note that these six grades are the only criterion used to decide whether a student can continue studying psychology. Even if they fail only one of these six examinations more than once, they are excluded from studying psychology at any Swiss university. This variable was dichotomized into pass and fail, as students must pass *all* six examinations to continue their study. Thus, examination grades are the only parameter to determine academic success in the first year at university. Further, to determine external validity of the examination grades, high school grades between students who failed the first year examinations were compared to students' high school grades who passed. Various studies have shown that high school grades are a strong predictor of academic performance [48] and high school performance has been used as a measure to determine scholastic aptitude in university students, when standardized tests are not available [21]. Participants who passed all six examinations reported significantly better average high school grades than participants who did not pass all six examinations, *M* = 4.84, *SD*  = 0.45 versus *M*  = 4.53, *SD*  = 0.39, respectively, where higher numbers represent better high school performance; *t*(66) = −3.12, *p* = .003.

#### Daily Questionnaire

*Daily sleep quality* was assessed with the item from the German version of the Pittsburgh Sleep Quality Index [49] which has been shown to have the highest correlation between a single-item and the final score of the full scale (single item – total correlation), *r* = 0.83 [50]. Specifically, participants rated their sleep quality during the previous night on a 4-point Likert scale from 1 (very bad) to 4 (very good).

*Physical activity* was evaluated with the Godin Leisure-Time Exercise Questionnaire [51], adapted to the daily online questionnaire format [34]. Participants were instructed to report their physical activity during the last 24 hours in terms of the number of minutes engaged in mild (minimal effort; e.g., easy walking), moderate (not exhausting; e.g., fast walking), and strenuous exercise (heart beats rapidly; e.g., running). The daily minutes of mild, moderate, and strenuous exercise were weighted by metabolic equivalents and then summed to produce a total daily leisure activity score. High scores reflect high levels of physical activity [51]. The original Godin Leisure-Time questionnaire has been found to have adequate concurrent validity with accelerometry and with maximum oxygen consumption [52].

*Daily positive and negative affect* were assessed with the German version of the pleasantness scale [53], a six-item instrument that has been shown to lead to comparable results as the Positive and Negative Affect Schedule (PANAS) [54] in a non-clinical sample [53]. Participants indicated the extent to which they had experienced each of the following six emotions during the last 24 hours on a 7-point Likert scale from 1 (not at all) to 7 (extremely): happy, content, cheerful, sad, downhearted, and frustrated. Internal consistency reliabilities were α = .95 (*M* = 13.35, *SD*  = 4.83) for positive affect and α = .87 (*M* = 7.37, *SD*  = 4.31) for negative affect calculated for all 32 measurement occasions.

As an additional measure of *academic performance* next to examination success, daily learning goal achievement was assessed on a day-to-day-level. Participants were asked whether they had achieved the learning goals they had set themselves for the previous 24 hours on a 5-point Likert scale from 0 (not at all) to 4 (completely) [22]. Importantly, examinations in the current study took place towards the end of the study period. Thus, examination success could not be used as a measure of academic performance during the entire period of studying towards the exams. Therefore, to understand the daily dynamics of academic performance during the study phase, learning goal achievement was measured daily, which is closely related to examination success [20].

### Statistical analyses

We used multilevel structural equation models, which are suitable for analyzing the nested structure of repeated measures data, and which handle missing data and varying time intervals in an appropriate way [55], [56]. We used a two-level model, with measurements nested within participants. Multilevel structural equation models, an extension to multilevel mediation models, have been shown to lead to unconflated estimates of between- and within-level components of indirect effects, thereby avoiding the biased estimates that can occur with more traditional multilevel models [55]. In multilevel structural equation models, person-mean centering is used by default. That is, the variables on the within-person level denote deviations from each individual's mean (e.g., individual learning goal achievement on days with high vs. low sleep quality). There is an implicit partitioning of the observed variables into latent within and between components. Here, we used a fixed slopes model in which only intercepts, but not slope parameters, were allowed to vary between individuals.

The mediation hypotheses on the between-person level as well as on the day-to-day level were analyzed in four distinct models, separately for the two predictors sleep quality and physical activity and for the two mediators positive and negative affect. Hence, each of our multilevel structural equation models contained one predictor, one mediator, and one outcome variable. This allowed us to test Hypotheses 1 and 2 for each predictor–mediator–outcome triplet (see Figure 1). We did not include all variables of interest within the same model, to avoid problems with overfitting [57]. Because examination success could be assessed only once at the end of the academic year, it was integrated in the between-person model, but could not be implemented in the within-person model, which is based on multiple measurements of each construct. To compare the results concerning the day-to-day dynamics of the within-person model with the average levels of the between-person model, we tested identical models on the within- and between-person level: Both models tested the relation between health behaviors, affect, and academic performance. In addition, examination success was added as outcome to the between-person model. To account for the expected nonlinear trend of learning goal achievement and affect over time, we included a temporal linear, quadratic, and cubic term in the model. As physical activity and negative affect were not normally distributed, they were transformed to approximate a normal distribution. We used Mplus (version 6.12) for the multilevel structural equation models and R (R version 2.15.1) for the remaining analyses.

## Results

Descriptive statistics of the variables of interest are presented in Table 1. In general, participants reported considerably high sleep quality and there was a large variability in physical activity. Participants also reported medium learning goal achievement as well as relatively high positive and relatively low negative affect. Forty-seven percent of the participants passed all six examinations.

### Between-person level

#### Hypothesis 1a: Association of sleep quality and physical activity with learning goal achievement (total effects)

Supporting our hypothesis, students with higher average sleep quality reported better learning goal achievement (*c* = 11.03, *SE*  = 1.35, *p*<.001; see Table 2 for all results). However, there was no significant association between physical activity and learning goal achievement (*c* = 0.30, *SE*  = 0.17, *p* = .079).

**Table 2:** Direct, total, and mediated effects of sleep quality and physical activity on learning goal achievement via positive and negative affect on a between-person level.

Predictor	Sleep quality Positive affect		Sleep quality Negative affect		Physical activity Positive affect		Physical activity Negative affect	
Mediator								
	*B* (*SE*)	*p*	*B* (*SE*)	*p*	*B* (*SE*)	*p*	*B* (*SE*)	*p*
*a*	19.84 (3.43)	<.001	−5.74 (1.08)	<.001	0.87 (0.17)	<.001	−0.19 (0.07)	.007
*b*	0.15 (0.05)	.007	−0.16 (0.19)	.385	0.30 (0.05)	<.001	−0.66 (0.19)	.001
*ab (indirect effect)*	2.91 (1.19)	.014	0.94 (1.09)	.385	0.27 (0.07)	<.001	0.13 (0.07)	.051
Mediated proportion (%)	26%		8.5%		88%		42%	
*c (total effect)*	11.03 (1.35)	<.001	11.05 (1.36)	<.001	0.30 (0.17)	.079	0.30 (0.17)	.081
*c' (direct effect)*	8.12 (1.81)	<.001	10.11 (1.90)	<.001	0.04 (0.18)	.839	0.17 (0.18)	.346

*Note*. Ratios are based on absolute values of direct and indirect effects, e.g. positive affect mediated 26% of the total effect between sleep quality and learning goal achievement. *a* = path from predictor to mediator; *b*  =  path from mediator to outcome;

*B*  =  unstandardized regression coefficient; *c*  =  *ab* (indirect effect) + *c’* (direct effect); *c’*  =  path from predictor to outcome after controlling for the mediator; *SE*  =  standard error.

#### Hypothesis 1b: Positive and negative affect as mediators (indirect and direct effects)

The relation between average sleep quality and average learning goal achievement was partially mediated by positive affect (see Table 2). In other words, sleep quality was positively related to positive affect, which was in turn positively related to learning goal achievement. The ratio of the indirect effect to the total effect was 26% for positive affect; that is, 26% of the effect of average sleep quality on learning goal achievement was mediated by positive affect. However, even when the mediator positive affect was included in the analysis, the relation between sleep quality and learning goal achievement remained significant, implying that additional factors also influence the relation between sleep quality and learning goal achievement. Negative affect, in contrast, did not significantly mediate the relation between sleep quality and learning goal achievement.

The relation between average physical activity and average learning goal achievement was mediated by positive affect (see Table 2). In other words, physical activity was positively related to positive affect, which in turn predicted learning goal achievement. Positive affect mediated 88% of the total effect between average physical activity and learning goal achievement. Negative affect did not mediate the relation between physical activity and learning goal achievement. Because of the gender differences in negative affect, all mediation models including negative affect were also tested excluding male participants (*n* = 8), leaving a sample of *n* = 64. Coefficients derived from these models were comparable with those based on the full sample (*N* = 72). We did not test separate models for male participants because too few men were included in the sample (*n* = 8), precluding reliable estimates from multilevel structural equation models.

#### Hypothesis 1c: Association between learning goal achievement and examination success

Learning goal achievement positively predicted examination success. For every 1-point increase in average learning goal achievement over the examination period, the odds of passing all six examinations increased by three and a half (OR  = 3.54, 95% CI: 1.42–8.81, *p* = .006).

### Day-to-day level (within-person level)

#### Hypothesis 2a: Association of day-to-day sleep quality and physical activity with daily learning goal achievement (total effects)

As shown in Table 3, on days with higher sleep quality, participants reported better learning goal achievement (*c* = 0.76, *SE*  = 0.33, *p* = .021). However, day-to-day physical activity was not significantly associated with learning goal achievement (*c* = −0.05, *SE*  = 0.03, *p* = .116).

**Table 3:** Direct, total, and mediated effects of sleep quality and physical activity on learning goal achievement via positive and negative affect on a within-person level.

Predictor	Sleep quality Positive affect		Sleep quality Negative affect		Physical activity Positive affect		Physical activity Negative affect	
Mediator								
	*B* (*SE*)	*p*	*B* (*SE*)	*p*	*B* (*SE*)	*p*	*B* (*SE*)	*p*
*a*	1.90 (0.57)	.001	−0.85 (0.17)	<.001	0.17 (0.04)	<.001	−0.05 (0.01)	.001
*b*	0.16 (0.03)	<.001	−0.46 (0.07)	<.001	0.17 (0.03)	<.001	−0.48 (0.07)	<.001
*ab (indirect effect)*	0.31 (0.09)	.001	0.39 (0.08)	<.001	0.03 (0.01)	<.001	0.02 (0.01)	.001
Mediated proportion (%)	41%		52%		27%		23%	
*c (total effect)*	0.76 (0.33)	.021	0.76 (0.33)	.021	−0.05 (0.03)	.116	−0.05 (0.03)	.117
*c’ (direct effect)*	0.45 (0.32)	.160	0.36 (0.32)	.260	−0.08 (0.03)	.010	−0.07 (0.03)	.015

*Note*. Ratios are based on absolute values of direct and indirect effects, e.g. positive affect mediated 41% of the total effect between sleep quality and learning goal achievement. *a* = path from predictor to mediator; *b*  =  path from mediator to outcome;

*B*  =  unstandardized regression coefficient; *c*  =  *ab* (indirect effect) + *c’* (direct effect); *c’*  =  path from predictor to outcome after controlling for the mediator; *SE*  =  standard error.

#### Hypothesis 2b: Daily positive and negative affect as mediators (indirect and direct effects)

When the two mediators, positive and negative affect, were included in separate models for each mediator, both mediated the within-person relation between sleep quality and learning goal achievement (see Table 3). Thus, on days with higher sleep quality, participants reported increased positive and decreased negative affect, which was in turn linked to better learning goal achievement. Forty-one percent of the effect of sleep quality on learning goal achievement was mediated by positive affect; 52% by negative affect. Note that the corresponding direct effects were no longer significant when either mediator was included in the analyses, suggesting full mediation.

Positive and negative affect both mediated the within-person relation between physical activity and learning goal achievement (see Table 3). Thus, higher physical activity was associated with higher positive and lower negative affect. In turn, positive affect was related to better learning goal achievement; negative affect to poorer learning goal achievement. Although, as reported above, the association between physical activity and learning goal achievement (total effect) was not significant, the association between physical activity and learning goal achievement was significantly negative when positive and negative affect were controlled (direct effect). The combination of these *negative* direct effects (physical activity on learning goal achievement controlled for positive or negative affect) and *positive* indirect effects (physical activity on affect; affect on learning goal achievement) suggests inconsistent mediation [58]. Because direct and indirect effects were of fairly similar magnitudes and opposite signs, they cancelled each other out, resulting in a nonsignificant total effect [59]. In cases of inconsistent mediation, Alwin and Hauser [60] suggested to use absolute values of the direct and indirect effects to calculate the ratio of the indirect effect to the total effect. Following this approach, we found that positive affect mediated 27%, and negative affect 23% of the total effect between physical activity and learning goal achievement.

## Discussion

This prospective longitudinal study examined how health behaviors were associated with academic performance during a stressful examination period, and tested whether affect underlies this relation. To our knowledge, this is one of the first studies to combine research on health behaviors, affect, and academic performance, and to consider the respective day-to-day dynamics over a period of time. The study has two main sets of findings: First, overall sleep quality predicted learning goal achievement, whereas overall physical activity was not related to learning goal achievement. Positive affect mediated the relation between both health behaviors and learning goal achievement. Negative affect, in contrast, did not mediate the relation between any of the two health behaviors and learning goal achievement. Importantly, overall learning goal achievement was a strong positive predictor of examination success. Second, in terms of day-to-day dynamics, on days on which participants reported higher sleep quality, they also reported better learning goal achievement. However, there was no significant relation between day-to-day physical activity and learning goal achievement. Day-to-day positive and negative affect both mediated the association between day-to-day sleep quality and learning goal achievement as well as day-to-day physical activity and learning goal achievement.

### Sleep (not physical activity) predicts better learning goal achievement

In accordance with our hypothesis, students with higher overall sleep quality reported better overall learning goal achievement. This finding is in line with previous research on the between-person level indicating that better sleep quality is associated with better academic performance and grades in adolescents and young adults [4], [6], [7]. Importantly, the present study also replicated these findings on a day-to-day level: On days with better sleep quality, participants also reported better learning goal achievement, indicating that they might have experienced immediate improvement in their learning goal achievement. These results imply that high sleep quality in general as well as just one night of better sleep during a stressful examination period might help young adults to better achieve their learning goals.

Concerning average physical activity over the 32-day period, our results showed no association between physical activity and learning goal achievement. This result differs from previous research conceptualizing habitual physical activity as a trait, which showed that more physically active adolescents have better grades [9], [10] and better academic performance [11]. Also, in contrast to our hypothesis, our results found no significant relation between day-to-day physical activity and learning goal achievement. One potential explanation for the missing association between physical activity and learning goal achievement in the present study might be that the assessment took place during the stressful examination period, in which time spent being physically active was not available for studying, which may thus have impeded learning goal achievement. Most previous studies were of cross-sectional design conducted during non-stressful periods [9]. Moreover, other studies on the between-person level have concentrated on the relation between physical activity and either cognitive performance [5], grades [9], [10], or overall academic performance [11], whereas this study focused on learning goal achievement, which is more of a motivational variable. Overall, our results suggest that physical activity in general as well as on the day-to-day level seems to have no impact on learning goal achievement during an intensive preparation and examination period.

### Affect as a mechanism underlying the relation between health behaviors and learning goal achievement

Average positive affect mediated the association between sleep quality and learning goal achievement, supporting our hypothesis. This finding extends previous research on the between-person level that has shown a positive relation between sleep quality and positive affect [30] and demonstrated that positive affect is related to better goal achievement and more favorable progress judgments [23]. However, to our knowledge, no previous research has tested the relation between sleep quality, affect, and learning goal achievement in a single model. Contrary to our hypothesis and previous research, negative affect did not mediate the effect of average sleep quality on learning goal achievement. Interestingly, on the day-to-day level, the association between sleep quality and learning goal achievement was mediated by both positive and negative affect, as predicted. This suggests that the overall negative affect experienced over the study period did not underlie the relation between sleep quality and learning goal achievement. Yet considering the day-to-day dynamics, on days with poor sleep quality, negative affect seems to be one of the mechanisms underlying the relation between sleep quality and learning goal achievement. Previous longitudinal studies have only tested parts of our model. For example, one study showed a positive relation between sleep quality and positive affect [31]. Another found positive affect to predict higher learning goals [22]. It seems plausible that current affect is used as input at the moment of judgments as suggested by the mood-as-information model [24]. For example, when participants in the current study were asked whether they had achieved today's learning goal, they may have consulted their momentary affect to judge the day's learning goal achievement. Positive affect would be indicative of high learning goal achievement, negative affect of low learning goal achievement. Mood is a particularly valuable source of information in the absence of regular formal performance feedback, as is the case during examination preparation periods, when students have to evaluate their learning outcomes and motivate themselves. This study is one of the first to suggest experienced affect as a mechanism potentially underlying the relation between day-to-day sleep quality and learning goal achievement, and its findings underline the importance of considering affect when investigating university students' health- and learning-related behaviors.

Concerning the relation between physical activity, positive affect, and learning goal achievement, we found that overall positive affect mediated the relation between overall physical activity and learning goal achievement. In contrast, negative affect was not an underlying mechanism. Similar to sleep quality, negative affect over the stressful study period did not seem to play a role in the relation of physical activity and learning goal achievement. Day-to-day higher physical activity was indirectly linked with better learning goal achievement through increased positive and decreased negative affect. In other words, the affective benefits of engaging in physical activity during a stressful examination period, which is positively related to learning goal achievement, do not outweigh the reduced learning goal achievement associated with physical activity (controlled for positive or negative affect). Our results thus suggest that being physically active or not during the preparation and examination phase seem to have comparable immediate benefits and costs. These divergent findings for the between- and within-person level emphasize the importance of understanding day-to-day dynamics in addition to average values, and help disentangle short- and long-term effects of physical activity on academic performance.

### Learning goal achievement predicts examination success

Importantly, learning goal achievement emerged as a strong predictor of passing all six examinations. These findings underline once again the importance of understanding behaviors that enhance learning goal achievement, such as sleep and physical activity.

### Limitations

The following limitations of the present study should be noted. First, all data were assessed by means of online self-report questionnaires, including examination grades. The sample included in this study represents about half of all first-year psychology students at the University of Basel in the respective academic year. Importantly, the grades of our sample do not differ from the grades of all psychology students in that academic year. Additionally, participation in the current study was voluntary and anonymous; compensation for study participation was independent of examination outcome. While this is no proof of accuracy of the reported data, it is nevertheless a good indicator that participants did report their actual grades. Assessing grades from the official institution of the university (and thus not providing full anonymity to the students) might have compromised the accuracy of participants' study responses and thus the study goals. Future research should also obtain data on physical activity and sleep quality from additional, behavioral measurement sources such as accelerometry. However, previous studies have shown that behavioral assessments and subjective ratings of physical activity are significantly related [52], [61].

Second, causal relations or disentangling the chronological order in which behaviors might have influenced each other cannot be inferred from the findings due to the observational study design. Importantly though, the directions of the hypotheses formulated were based on theoretical considerations and previous research. Third, due to the prospective nature of the study, 12% of the sample dropped out. This is a low dropout rate. However, to evaluate whether the study sample differs in academic performance of all first-year psychology students at the University of Basel, we compared our sample's examination pass rate with that of all first-year psychology students at the University of Basel during the academic year in which the study took place and found no difference in their grades. Fourth, while the naturalistic design of this study over a stressful examination period is an important strength, it is also a limitation in that it does not allow the results to be generalized to other periods of the university year. Moreover, the study population is limited to psychology students of the University of Basel, meaning that the results cannot necessarily be generalized to students of other departments or universities.

Furthermore, mental disorders and stress have been shown to be related to health behaviors and experienced affect [26], [62]. While in the present study population only three students reported clinically relevant symptoms on the Beck Depression Inventory [63], other mental disorders were not assessed and might have affected the results. However, results of the analyses disregarding the three participants reporting clinically relevant symptoms on the Beck Depression Inventory were comparable to those including the full sample. Finally, in addition to sleep and physical activity, future research should also include day-to-day dynamics of other health behaviors, which might be relevant to affect or academic performance, such as eating behavior or alcohol consumption [64], [65].

## Conclusion

This study shed light on how two health behaviors—sleep quality and physical activity—are associated with affect and academic performance during a stressful examination period and highlighted the potential of these behaviors to promote young adults' academic performance. Notably, sleep quality was a stronger predictor of learning goal achievement than was physical activity on average but also on a day-to-day level. The findings extend the existing body of literature by integrating research on health behaviors, affect, and academic performance during a stressful examination period. Additionally, the results emphasize the importance of understanding within-person variability in sleep, physical activity, affect, and learning goal achievement in daily life. Designs such as that used in the present study make it possible to disentangle short- and long-term effects. For example, the present results showed that negative affect experienced over the study period did not underlie the relation between both health behaviors and learning goal achievement, however, on a daily measurement level, negative affect did mediate the relation between both sleep quality and physical activity with learning goal achievement. These findings provide important insights into what effects to expect, for example, in the process of a prevention or intervention program.

The within-person variability of health behaviors, learning goal achievement, and their underlying mechanisms is an important area for future research. This study is a first step toward a better understanding of the role of health behaviors for affect experience and academic performance during a demanding examination period at university. The findings can provide a basis for potential prevention and intervention programs that might allow more young adults to complete their first year at university in good physical and mental health as well as achieving their academic goals.
